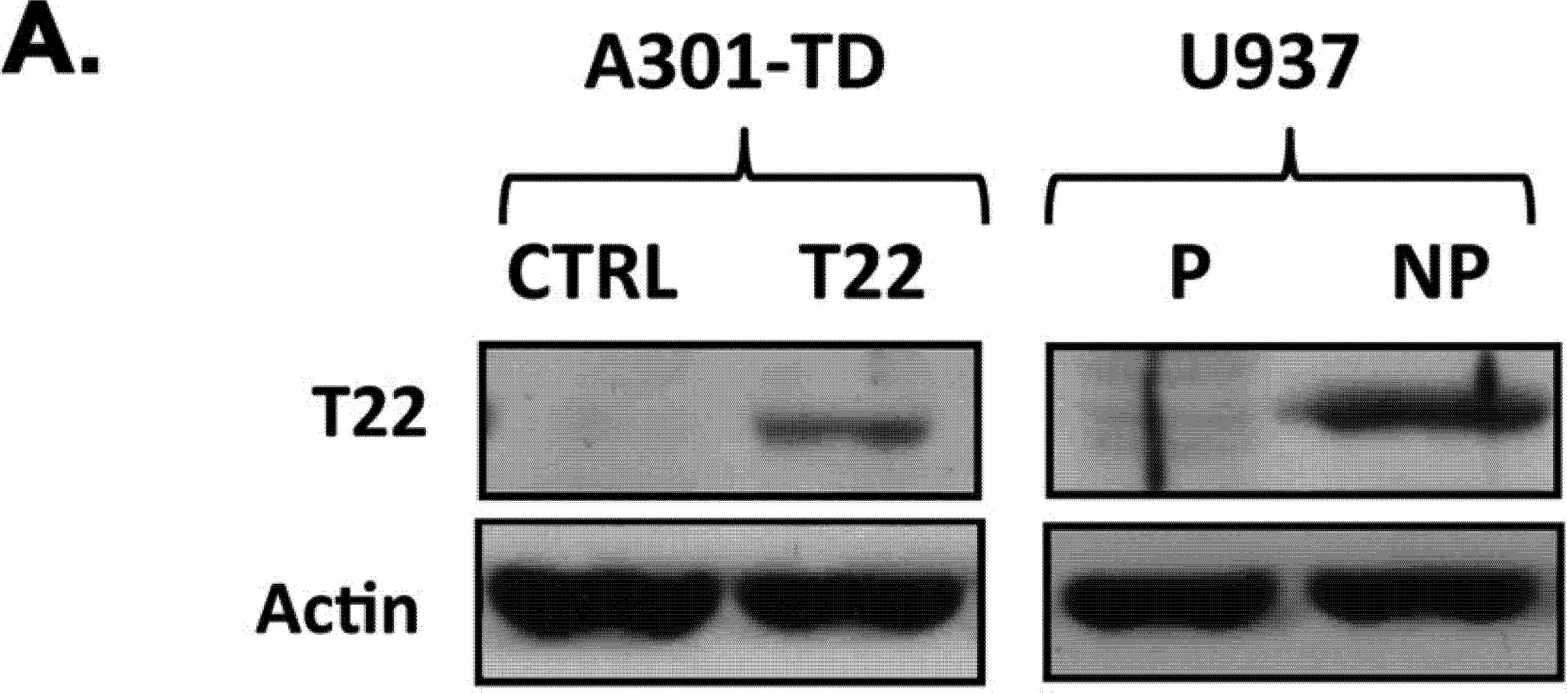# Correction for Kajaste-Rudnitski et al., “TRIM22 Inhibits HIV-1 Transcription Independently of Its E3 Ubiquitin Ligase Activity, Tat, and NF-κB-Responsive Long Terminal Repeat Elements”

**DOI:** 10.1128/jvi.02071-24

**Published:** 2025-01-27

**Authors:** Anna Kajaste-Rudnitski, Sara S. Marelli, Cinzia Pultrone, Thomas Pertel, Pradeep D. Uchil, Nadir Mechti, Walther Mothes, Guido Poli, Jeremy Luban, Elisa Vicenzi

## AUTHOR CORRECTION

Volume 85, no. 10, p. 5183–5196, 2011, https://doi.org/10.1128/jvi.02302-10. Page 5189: Figure 6A should appear as shown in this correction. In the original article, there was an erroneous duplication in the actin loading controls for the A301-TD cells. The error does not impact or alter the conclusions of the paper.

**Fig 6:**